# Observation of an atomic exchange bias effect in DyCo_4_ film

**DOI:** 10.1038/srep18377

**Published:** 2015-12-17

**Authors:** Kai Chen, Dieter Lott, Florin Radu, Fadi Choueikani, Edwige Otero, Philippe Ohresser

**Affiliations:** 1 Synchrotron SOLEIL, L’Orme des Merisiers, Saint-Aubin-BP48, 91192 GIF-sur-YVETTE CEDEX, France; 2 Institute for Materials Research, Helmholtz-Zentrum Geesthacht, 21502 Geesthacht, Germany; 3 Helmholtz-Zentrum Berlin für Materialien und Energie, Albert-Einstein-Str.15, 12489 Berlin, Germany

## Abstract

The fundamental important and technologically widely employed exchange bias effect occurs in general in bilayers of magnetic thin films consisting of antiferromagnetic and ferromagnetic layers where the hard magnetization behavior of an antiferromagnetic thin film causes a shift in the magnetization curve of a soft ferromagnetic film. The minimization of the single magnetic grain size to increase the storage density and the subsequent demand for magnetic materials with very high magnetic anisotropy requires a system with high **H**_**EB**_. Here we report an extremely high **H**_**EB**_ of 4 Tesla observed in a single amorphous **DyCo**_**4**_ film close to room temperature. The origin of the exchange bias can be associated with the variation of the magnetic behavior from the surface towards the bulk part of the film revealed by X-ray absorption spectroscopy and X-ray magnetic circular dichroism techniques utilizing the bulk sensitive transmission and the surface sensitive total electron yield modes. The competition between the atomic exchange coupling in the single film and the Zeeman interaction lead to an intrinsic exchanged coupled system and the so far highest exchange bias effect **H**_**EB**_** = 4** Tesla reported in a single film, which is accommodated by a partial domain wall formation.

Exchange bias effect (EB) was discovered in 1956 by Meiklejohn and Bean when studying Co particles embedded in their native antiferromagnetic oxide^1^. It is generally considered to form from an uncompensated spin configuration at the ferromagnetic/antiferromagnetic (FM/AF) interface^2^,^3^, as it is the case in small particles, inhomogeneous materials, FM films on AF single crystals and FM on AF thin films^4^ with frozen and rotatable spins at their interfaces^5^,^6^,^7^,^8^,^9^. Phenomenologically, the EB effect in FM/AF systems displays a shift of the hysteresis by an EB field H_EB_ that is achieved by a magnetic field cooling procedure down to the Néel temperature T_N_ of the AF. The experimentally observed value of the H_EB_ in FM/AF systems, however, is in general several orders of magnitude below the theoretical prediction for a perfect EB system^10^. This discrepancy resulted in a heavy debate and the development of sophisticated models for the explanation of the origin of the EB effect and its drastic reduction of the EB effect in real EB systems^4^,^11^,^12^. Besides the classical system of AF/FM interfaces, EB and related effects have been observed also in other types of samples, e.g. involving ferrimagnets (FI): AF/FI^13^, FI/FM^14^,^15^,^16^ and lately also in FI/FI^17^ with a compensated spin structures at the interface. Transition Metal-Rare Earth (TM-RE) alloys, in particular, are nowadays suggested to used as FI materials in magnetic hybrid structures exhibiting strong EB effects^18^.

Besides an interfacial exchange between two chemically and magnetically different compositions, a physically induced magnetic phase deviation from the bulk to the surface may also results into an exchange bias effect although there is no obvious chemical interface in the sample. The lower atomic coordination of the atoms at the surface results in a band narrowing, leading to changes of the moments at the surface in respect to the bulk. In MnF_2_ 19,20, it requires a lower magnetic field value to force the phase transition from an AF to a spin-flop state at the surface. Ferromagnetism on the surface of the bulk paramagnetic intermetallic compound YCo_2_ was predicted using full-potential electronic structure calculations^21^ and observed by spin-resolved photoelectron spectroscopy^22^. For rare earth materials, surface enhanced magnetic ordering have been found in Gd^23^ and Tb^24^ resulting in an increase of the Curie temperature for the surface states. Following the theoretical calculations performed by Camley *et al.*^25^, the surface phase transitions are responsible for the twisted magnetic spin structures observed in Fe/Gd multilayer^26^,^27^,^28^,^29^,^30^.

Figure 1 shows a schematic sketch of a single ferrimagnetic film system with the potential of intrinsic exchange bias effect. Surface and bulk of the film are of the same composition consisting here of two magnetic sites that are strongly exchanged coupled to each other but possess opposite signs in their resulting moments. If the surface and bulk parts are again exchanged coupled to each other, their different magnetic behavior in respect to an exernal applied magnetic field may lead to an competition between the Zeeman and the exchange coupling energy that potentially creates an EB system without the necessity of an additional magnetic layer. When no magnetic field is applied, the magnetization behavior of the surface layer follows the one of the bulk due to the exchange coupling, marked as II and III. When an sufficient high external magnetic field is applied, the Zeeman energy term will dominate for the surface layer and turn its resulting moment along the applied magnetic field direction. Thus state I or IV forms with an out-of-plane domain wall (DW) of a length of *ξ*. With respect to a classical EB system, there is no chemical interface in the EB system and therefore the EB effect is expected to be mediated by the formation of a domain wall. Since there is no contamination, pinhole effect and interfacial roughness that may reduce the EB strength, such a system has the potential to show large EB values.

In this paper we report the largest so far observed EB value measured in a single amorphous RE-TM alloy consisting of Dy and Co with a chemical composition of DyCo_4_. In this strongly atomically exchanged coupled system the surface and the rest of the film exhibit clear differences in their magnetic behavior with magnetic field and temperature. At a temperature of T = 270 K, the exchange coupling between the surface and the bulk states leads to the emergence of an additional asymmetric magnetic hysteresis loop with its center shifted by about *μ*_0_H_EB_ = 4T. Such a giant EB is observed for the first time originating from the atomic exchange coupling between a virtual surface part and the bulk part in DyCo_4_ without a chemical interface. This may be useful for creating novel magnetic exchange bias systems for future magnetic storage application by compacting several layers of a spin-valve system into a single one.

## Samples and Experimental set-up

The DyCo_4_ samples were prepared by magnetron sputtering (MAGSSY chamber at BESSY) in an ultraclean argon atmosphere of 1.5 × 10^−3^ mbar with a base pressure of p < 5 × 10^−9^ mbar at the deposition temperature of 300 K. The stoichiometry of the ferrimagnetic alloys was controlled by setting the deposition rate of separate chemical elements in a co-evaporation scheme. Si_3_N_4_ membranes with a surface area of 5 × 5 mm^2^ and a thickness of 100 nm were used as substrates for the soft x-ray transmission measurements. A capping layer of 3 nm of tantalum(Ta) was grown on the 50 nm thick DyCo_4_ layer to prevent surface oxidation. As a capping layer to prevent oxidation, Ta layer have been used and commonly considered to have no influence to the macroscopic magnetism of the layer underneath^17^,^31^. However, In our case we observe a rather large difference between the surface and the bulk XMCD spectra. We believe that this large difference is caused mainly by noncollinearity (conical-like) in the magnetic structure of DyCo_4_. Also, considering the ferrimagnetism of DyCo_4_, the surface atoms have different chemical environments and the exchange coupling energy between Dy and Co may be smaller due to the reduced coordination number. As a result, these two effects may concur contributing to the observed differences between the bulk and the surface magnetism.

**Figure 2:**
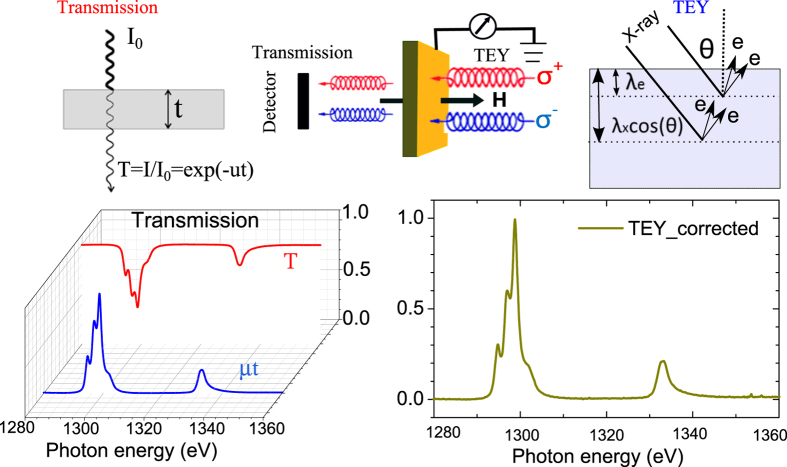
Experimental setup for surface and bulk magnetism. (Up)XMCD measurements of transmission and TEY mode, the transmitted x-ray intensities and total electron yield from the sample are recorded. (Down)The XAS was extracted as the logarithm of the ratio of the transmitted x-ray intensities, or the drain current from the sample after the correction of the saturation effect.

X-ray absorption and XMCD spectra at the Co L_2,3_ and Dy M_4,5_ edges were measured at temperatures from 4.4 K to 300 K in transmission geometry at the synchrotron facility SOLEIL using the 7T cryomagnet installed on the DEIMOS (Dichroism Experimental Installation for Magneto-Optical Spectroscopy) beamline^32^,^33^. The XMCD studies were carried out using x-rays of circular right (CR) and left (CL) polarizations with a magnetic field of *μ*_0_H = 6T applied along the x-ray beam (see Fig. 2). For the magnetic sensitivity to the individual magnetic elements, the characteristic energies for the Co L_2,3_ and the Dy M_4,5_ edges were selected. The XMCD data were recorded as (*σ*^+^ − *σ*^−^), where *σ*^+^ and *σ*^−^ denote the absorption cross section for CR and CL polarized x-rays, respectively. The magnetic hysteresis measurements were performed by taking the difference of (*σ*^+^ − *σ*^−^) with the photon energy for the x-ray beam set to the value at the L_3_ edge of Co or M_5_ edge of Dy where the XMCD signal is maximized. For the transmission measurements^34^, the experimental XMCD is extracted as the logarithm of the ratio of the transmitted x-ray intensities of different polarization states and probes the whole sample. The total electron yield (TEY) signal was recorded at the same time giving a high surface sensitivity with a sampling depths λ_e_ of ~2 nm^35^,^36^. As long as the sampling depth remains much shorter than the x-ray attenuation length λ_x_cos(*θ*) (with *θ* = 0 in our experiments), the TEY signal represents the true XAS and XMCD with the accuracy of ~5 − 10%^35^,^37^ compared to the results after the correction of the saturation effects. The combination of both methods allows one to separate the surface from the bulk contributions.

## Experimental results

In amorphous Dy_x_Co_100−x_ the magnetic configuration is determined by the competition between the magnetic atomic exchange interaction and the Zeemann interaction. The latter favors the Co and the Dy magnetic moments being aligned both in the direction of the applied magnetic field, while the atomic exchange interaction is minimized if Dy and Co atoms are in an antiferromagnetic configuration. The strong exchange interaction in Dy_x_Co_100−x_ between the sites forces them in general into an AF alignment while their resulting moment is turned into the direction of the applied magnetic field. One of the intriguing properties of amorphous systems consisting of two magnetic different sites belonging to the RE and TM series, respectively, is the different temperature dependence of their magnetic moment resulting into a change of the magnetically dominant site. In Dy_x_Co_100−x_, Dy possesses at low temperatures a much larger magnetic moment compared to the one of Co. On the other hand, the Dy moment decays much stronger with increasing temperature until the compensation temperature T_comp_ is reached when the total magnetic moment of both sites are equal. At temperatures higher than T_comp_ the compound enters into the Co dominant phase accompanied with a reversal of the orientation for both sites due to the strongly AF exchanged coupling between both magnetic sites. T_comp_ depends on the individual composition and can be controlled by the composition parameter x, and is expected to be ~250 K for DyCo_4_.

**Figure 3:**
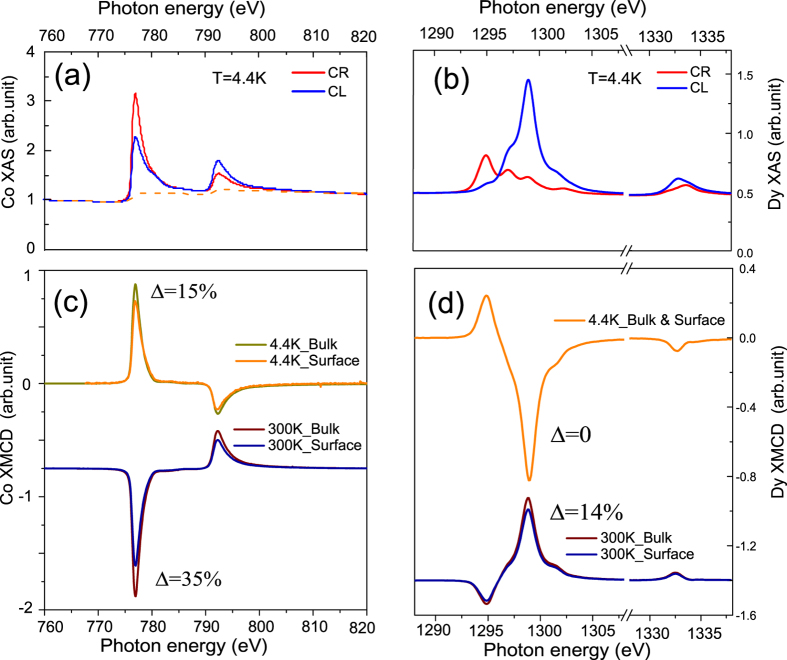
XAS and XMCD of Co and Dy. XAS at the Co L_2,3_ (**a**) and Dy M_4,5_ (**b**) with the magnetic field of *μ*_0_H = 6T applied out of plane at T = 4.4 K. Surface and bulk XMCD of Co (**c**) and Dy (**d**) at T = 4.4 K and 300 K. For Co, the surface XMCD signal is ~15% and 34% reduced compared to the bulk at 4.4 and 300 K, respectively. For Dy, the surface XMCD signal is the same or 14% reduced compared to the bulk at 4.4 or 300 K, respectively. A more reduced Co surface magnetism at high temperature is observed compared to that of Dy.

X-ray absorption spectroscopy (XAS) and XMCD measurements were carried out at the Co L_2,3_ and the Dy M_4,5_ edges for a series of temperatures, utilizing the bulk sensitive transmission and the surface sensitive total electron yield (TEY) modes. The spectra for T = 4.4 K from transmission measurements taken at an applied magnetic field of *μ*_0_H = 6T are shown for the Co and Dy site in Fig. 3(a,b), respectively. The resulting surface and bulk XMCD spectra of Co and Dy at *μ*_0_H = 6T are displayed in Fig. 3(c,d) for T = 4.4 K and 300 K. The positive (negative) XMCD signal of Co (Dy) at 4.4 K indicates here that the out of plane projection of the magnetic moment 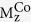 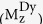 is antiparallel (parallel) aligned along the applied magnetic field as expected for the Dy dominated magnetic state at low temperatures. At T = 300 K a clear change of the sign in the XMCD signal at both absorption edges is observed indicating that the out of plane projections of the magnetic moments 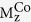 and 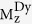 have reversed their direction and DyCo_4_ with a compensation temperature below is now in the Co dominated state. Interestingly, the bulk XMCD signal for Co is about 15% higher in magnitude at 300 K compared to that of 4.4 K, while for Dy the XMCD signal is about 45% reduced in magnitude for the higher temperature. The reduction of the moment at low temperature may be explained by the formation of a cone structure at low temperatures as it is observed in other rare earth materials as e.g. Ho^38^ or in similar RECo_5_ compounds^39^. At higher temperatures, the decrease of the XMCD signal for the Dy site indicates the decrease of the Dy moment with temperature. For both temperatures a clear deviation between the XMCD signal from the TEY and the transmission measurements is observed for the Co site. The surface sensitive XMCD signal at the Co edge from TEY is ~15% and 34% reduced compared to that bulk sensitive XMCD from transmission at T = 4.4 K and 300 K, respectively. For the Dy site the effect is much smaller. At 4.4 K, no significant differences can be observed, while at 300 K the surface moments is only 14% reduced compared to bulk. It should be noted that the TEY data is already corrected for the reduction of the signal due to saturation effects by ~5 − 10%^35^,^37^. Since TEY probes only the first few nanometers (~2 nm) at the surface and the transmission measurement is sensitive to the magnetism of the complete film with a thickness of 50 nm with the surface magnetism giving only a tiny contribution of ~4%, it can be concluded that the magnetism at the surface (denoted as the surface part) differs significantly from the rest of the film (denoted here as the bulk part).

**Figure 4:**
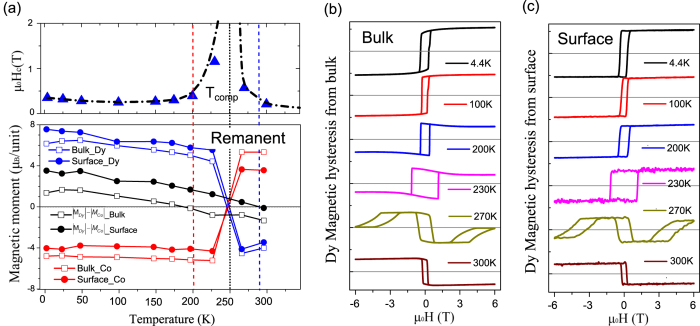
Temperature dependent magnetic properties of Dy and Co. (**a**) (up) Coercivity *μ*_0_H_c_ from the temperature dependent magnetic hysteresis loops and (down) the remanent bulk and surface magnetic moments of Dy and Co, respectively. The values of 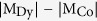 of the bulk and surface are also listed showing significant differences. The error bar from the sum-rule calculations (~%10) are not shown, the statistic error bar (~%4) are smaller than the size of the points. Panels (**b**,**c**) show the temperature dependent (4.4–300 K) Dy magnetic hysteresis loops for the Dy site taken by XMCD measurements for the bulk and the surface parts, respectively.

By the application of the sum rules^34^,^40^,^41^ for the measured XAS and XMCD spectra, the bulk magnetic moments of Dy and Co are determined to 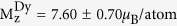 and 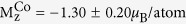 at T = 4.4 K, and 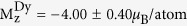 and 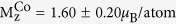 at 300 K, respectively. The magnetic dipole term *T*_*z*_ associated with the spin-dependent asphericity of the electronic cloud can be neglected for Co^34^, but not for the rare earth element Dy^41^ where it can be approximated as 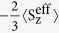. One should note that the XMCD signals (*σ*^+^ − *σ*^−^) as well as the magnetic moments for Co and Dy have opposite signs at both temperatures, demonstrating their antiparallel alignment. At T = 4.4 K, the magnetic configuration is in the Dy dominant phase in which 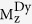 is pointing along the direction of the applied magnetic field while at T = 300 K, the situation is reversed in the Co dominant phase with now 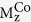 aligning along the magnetic field direction. At low temperatures as T = 4.4 K Dy possesses a magnetic moment as high as 10*μ*_B_/atom with J = 15/2. The significantly lower value found here in the alloy indicates that the magnetic moments of Dy are not completely aligned along the applied magnetic field direction since the XMCD method is only sensitive to the moments projection along the magnetic field direction. This may be explained by the formation of a cone spin structure of Dy as already mentioned before and account also for the reduction of 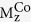 at low temperatures due to the strong antiferromagnetic coupling between Co and Dy. Temperature dependent magnetic hysteresis loops were recorded at the photon energies where the XMCD signals for both elements Dy (E = 1298.4 eV) and Co (E = 778 eV) were largest. Figure 4 shows the temperature dependent hysteresis loops recorded at the Dy absorption edges in the bulk sensitive transmission (Fig. 4(b)) and in surface sensitive TEY mode (Fig. 4(c)). The hysteresis loops taken at the Co absorption edge (see SI) follow the Dy hysteresis loops demonstrating the antiparallel exchange coupling between both elements in the alloy for all temperatures. To determine the compensation temperature at which the total magnetic contributions from Dy and Co are equal, i.e. when the net magnetic moment of the alloy becomes zero, the coercivity *μ*_0_H_c_ is plotted as a function of temperature shown in Fig. 4(a). The steep increase and decrease of the value of the coercivity *μ*_0_H_c_ at 230 K and 270 K, respectively, can be fitted by exponential functions. By the determination of their common asymptote, T_comp_ can be estimated to about 250 K. The lower panel of Fig. 4(a) shows the bulk and surface magnetic moments of Dy and Co, respectively, for various temperatures from 4.4 K to 300 K at remanence calculated by the sum rules. For both parts, the total moments for Dy and Co undergo a change of their magnetization direction in respect to the applied magnetic field at around 250 K as it is expected at T_comp_. The resulting magnetic moments, calculated by taking the difference of the total magnetization for both sites, 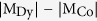, however, show significantly deviations from the overall determined T_comp_. The resulting moment for the bulk part already reverses its sign at ~200 K, while the surface part remains in the Dy dominant phase up to ~290 K demonstrating a clear magnetic phase separation between the surface and the bulk state of the film in this temperature regime.

By using the transmission and TEY mode, the temperature dependent magnetization behavior is surveyed for the bulk and surface state of the film separately as it is shown in Fig. 4(b,c), respectively. For both series of hysteresis loops the temperature dependent transition from the dominate Dy to the dominate Co magnetic state is observed, however, the saturation behavior shows clear differences in the bulk and surface part, particularly around the compensation temperature. The bulk magnetization 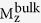 exhibits at T = 200 K a clear reduction at higher magnetic fields that becomes even more pronounced by approaching the compensation temperature. The surface part, on the other hand, displays a square shape of the hysteresis loops that remain unchanged even close to the transition temperature. Such differences in the behavior of bulk and surface magnetization indicates the formation of a domain wall with a partial winding that takes place deeper in the sample.

Particularly interesting is the magnetic behavior at a temperature slightly higher than the compensation temperature, when the film is supposed to enter into the Co dominated magnetic state. As expected for a change from the Dy to Co dominant state, the remanent magnetization flips its sign at T = 270 K compared to the situation below T_comp_. On the other hand, additional “wing shape” hysteresis loops can be observed when 6T > *μ*_0_H > 2T and −6T < *μ*_0_H < −2T. At *μ*_0_H = 6T, 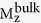 is almost zero while 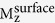 reverses its magnetization direction to a non-zero value. Cycling the magnetic field between 6T > *μ*_0_H > 0T or −6T < *μ*_0_H < 0T, results into an asymmetric hysteresis loop with two magnetically distinct states. Considering the definition of the exchange bias field H_EB_ as a shift of the center of the hysteresis loop from zero field, we observe here a extremely high H_EB_ of around 4T. The cycling between these high and zero fields show that the shape of the loops and H_EB_ remains constant without observable training effects.

## Discussion

**Figure 5:**
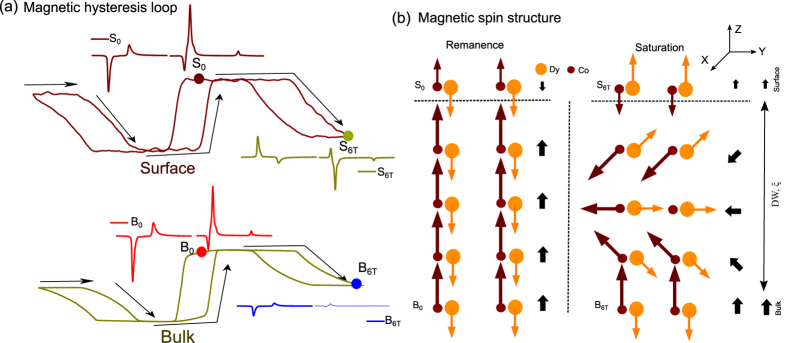
Magnetic spin configuration at T = 270 K. (**a**) Surface and bulk magnetic hysteresis loops of Dy at 270 K. XMCD of Dy and Co at the 4 states of S_0_, S_6T_, B_0_ and B_6T_ marked in the hysteresis are also shown. With *μ*_0_H = 6T, opposite magnetization from the surface to the bulk is observed. (**b**) Sketch of the spin structure of DyCo_4_ at 270 K for the remanant and saturation state with an out of plane magnetic domain wall from the surface to the bulk.

In order to understand the peculiar behavior at T = 270 K with its addition wing shape hysteresis loops at higher magnetic fields, the individual magnetic behavior of the surface and the bulk part as well as their interplay have to be considered. For low magnetic fields, when the Zeeman interaction is weak and the exchange interaction is the dominating term, it is energetically favorable for the system that the Dy dominant surface part follows the bulk magnetism that is already in the Co dominant state. The system remains magnetically stable up to approximately *μ*_0_H = 4T, when the Zeeman interaction becomes finally strong enough to compete with the exchange interaction of the system resulting into a rotation of the magnetically strongly coupled Dy and Co in the surface part with 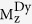 aligning along the applied magnetic field direction. For the bulk configuration, on the other hand, the reversal of the surface magnetization leads to a domain wall configuration to store the exchange energy which is release back when the Zeeman energy is reduced again analogue to the situation just below T_comp_. This is reflected in the reduction of the magnetization projection 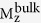 for Dy and Co driven by the surface magnetization as indicated by the correlated hysteresis loops between surface and bulk parts. Figure 5(b) shows schematically the magnetic configurations in the film for the two distinct states in the wing hysteresis. In the lower magnetic state, the resulting moment is determined by 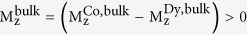 while 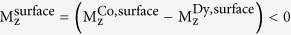. At this state, 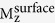 aligns antiparallel to the magnetic field due to the exchange coupling with the bulk part, as the identical signs in the XMCD signals in Fig. 5(a) reveal.

In the higher magnetic state resulting 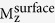 aligns now parallel along the field direction while 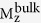 is reduced due to the formation of a complex domain winding state. The occurrence of the wing shape hysteresis loops shows that the surface and bulk states of the film are strongly magnetically coupled by the atomic exchange interaction keeping the two magnetic states stable in a certain magnetic field range. At high fields, from the interface to the bulk, an out of plane domain wall is formed with the wall energy *σ*_*w*_ stored in the boundary. According to the Mauri’s model^11^, the atomic exchange field can be described as: 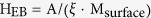, where A is the atomic exchange stiffness, *ξ* is the thickness of the domain wall as shown in Fig. 5(b) and M_surface_ is the surface magnetization. At low magnetic fields the magnetically softer bulk part determines the magnetism of the system while at higher magnetic fields the magnetically harder surface layer becomes the trigger of the change in the magnetic configuration. Since here the atomic exchange between both sides is crucial for the observed phenomena of the huge shift of the wing shape hysteresis loop, this effect is named atomic exchange bias effect.

It is reasonable that an out-of-plane magnetic domain wall expands throughout the film of 50 nm. According to Mauris model, the domain wall width can by estimated according to the following relationship: 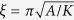, with A and K being the exchange stiffness and exchange anisotropy. Assuming the parameter A and K for DyCo_4_ to be similar to that of TbFe at 300 K 42, which are A = 8 * 10^−8^ erg/cm and K = 2.8 * 10^4^ erg/cm^3^, the resulting domain wall equals to ~53 nm.

In conclusion, a huge exchange bias effect with H_EB_ was observed in a single DyCo_4_ alloy film by using synchrotron soft x-ray magnetic circular spectroscopies at the Dy M_4,5_ and Co L_2,3_ edges in transmission and TEY mode. It was found that the magnetism on the surface behaves drastically different as in the bulk of the DyCo_4_ film. At T = 270 K just above the T_comp_, the bulk magnetism is dominated by the Co cite while the surface magnetism is still in the Dy dominant state due to the strong reduction of the Co magnetization at the surface. The strong atomic exchange interaction couples the surface and bulk part leading to two magnetic stable states that can be observed as wing shape hysteresis loops at high magnetic fields, which also exhibits an asymmetric behavior. Such a giant exchange bias effect of up to *μ*_0_H_EB_ = 4T is reported for the first time for a single film without an additional chemical interface. The availability of large exchange bias effects may be of great importance for future magnetic data storage devices for making use of materials with extremely high magnetic anisotropy.

## Additional Information

**How to cite this article**: Chen, K. *et al.* Observation of an atomic exchange bias effect in DyCo_4_ film. *Sci. Rep.* **5**, 18377; doi: 10.1038/srep18377 (2015).

## Supplementary Material

Supplementary Information

## Figures and Tables

**Figure 1:**
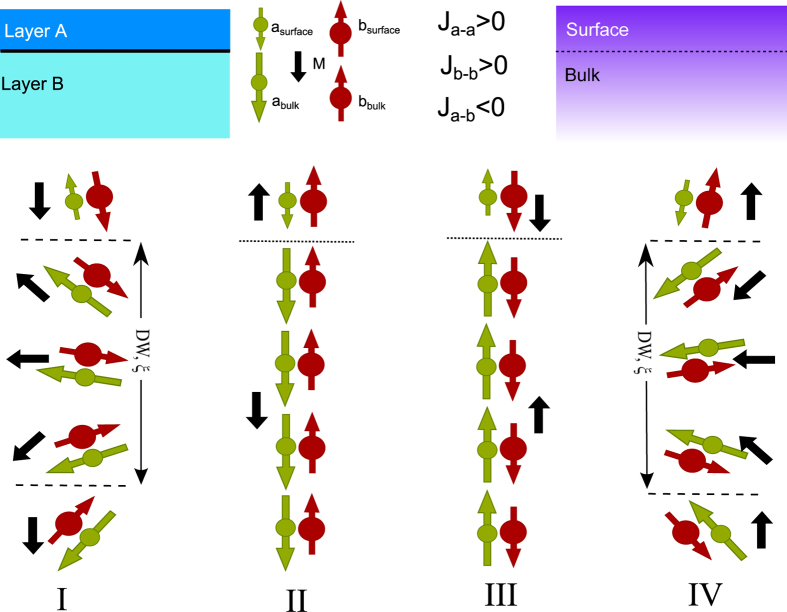
Sketch of the exchange bias system from single layer with different surface and bulk magnetism. The classical bilayer model is also shown for a better understanding. Both the layer A and B are ferrimagnetic materials consisting of antiparallel coupled atoms of Dy and Co. The layers A (or surface) and B (or bulk) are dominated by Dy and Co, respectively. There exist four different magnetic phases marked as I, II, III and IV with different magnetic field, with out of plane domain wall formed in I and IV.
