# Supplementary material for: Observation of an atomic exchange bias effect in DyCo4 film
Source: Sci Rep. 2015 Dec 17;5:18377. doi: 10.1038/srep18377 (PMC4682085; doi:10.1038/srep18377)
Supplement: Supplementary Information [file srep18377-s1.pdf]

Supplemental Materials: Observation of an atomic exchange bias effect in $DyCo_4$ film

Kai Chen[1,*], Dieter Lott[2], Florin Radu[3], Fadi Choueikani[1], Edwige Otero[1], and Philippe Ohresser[1]

[1]Synchrotron SOLEIL, L'Orme des Merisiers,Saint-Aubin-BP48, 91192 GIF-sur-YVETTE CEDEX,France [2]Institute for Materials Research, Helmholtz-Zentrum Geesthacht, 21502 Geesthacht, Germany [3]Helmholtz-Zentrum Berlin für Materialien und Energie, Albert-Einstein-Str.15, 12489 Berlin, Germany

1:MAGNETIC MOMENTS

X-ray absorption and XMCD spectra, measured with circular left (CL) and right (CR) polarized beam, at the Co $L_{2,3}$ and Dy $M_{4,5}$ with T=4.4K and 300K are listed in Fig1(a),(b),(c),(d) with an out of plane field of μ_0H = 6T, together with the integration of the XAS and XMCD in Fig1(e), (f), (g) and (h). With the temperature increasing from 4.4K to 300K, the XMCD signal for Co exhibits a change from positive to negative. The XMCD signal at 300K is about 15% higher in magnitude compared to that of 4.4K. For Dy, at 4.4K and 300K, the magnetic projection are parallel or antiparallel to the field according to their negative or positive XMCD signal, respectively. Furthermore, the XMCD signal at 300K is about 45% reduced in magnitude compared to that

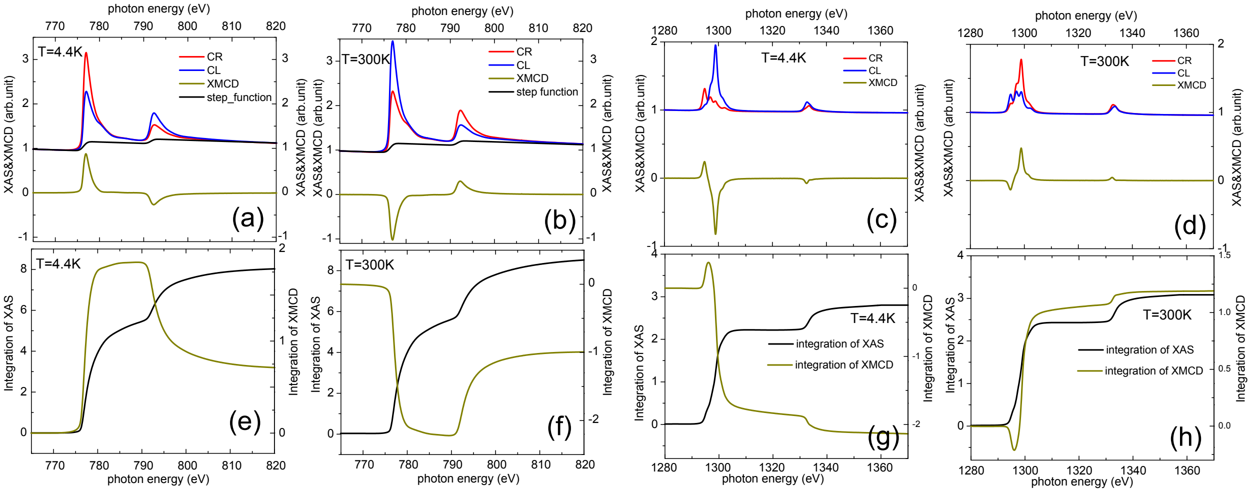

FIG. 1. (color Online) X-ray absorption and XMCD spectra at the Co $L_{2,3}$ and Dy $M_{4,5}$ at T=4.4K and 300K are listed in(a),(b),(c) and (d) with an out of plane field of μ_0H = 6T, together with the integration of the XAS and XMCD in (e),(f), (g) and (h).

recorded at 4.4K. For Dy, the shape of XAS undergoes a considerable change with temperature, as revealed by the occupation of the Zeeman split 4f states, denoted as M_J states [4]. This reshuffle of the different sub-states is accompanied with a strong decrease of the magnetic moment at the Dy site as it can be clearly seen in the reduction of the XMCD signal at T=300K. By applying the sum rules [1–3], which are

$$\frac{\int_{edge} dw(\mu^+ - \mu^-)}{\int_{edge} dw(\mu^+ + \mu^- + \mu_{//})} = \frac{1}{2}\frac{c(c+1) - l(l+1) - 2}{l(l+1)(4l+2-n))} M_L \tag{1}$$

$$\frac{\int_{j_+} dw(\mu^+ - \mu^-) - [(c+1)/c]\int_{j_-} dw(\mu^+ + \mu^-)}{\int_{j_+ + j_-} dw(\mu^+ + \mu^- + \mu_{//})} = \frac{1}{2}\frac{l(l+1) - c(c+1) - 2}{3c(4l+2-n))} M_S^{eff} \tag{2}$$

it is possible to determine the effective spin magnetic moment m_S^{eff} and the orbital magnetic moment m_L. For Co $L_{2,3}$ we have c = 1 and l = 2 while for Dy $M_{4,5}$ we have c = 2 and l = 3. Here, n or $(4l + 2 - n)$ are the numbers of electrons or holes in Co 3d and Dy 4f states, respectively. The effective spin moment $M_s^{eff} = M_S - C \cdot m_T$ (C=7 for Co and 6 for Dy) consists of the spin magnetic moment m_s and the magnetic dipole moment m_t which accounts for the asphericity of the spin moment distribution. The integration of the XAS and XMCD at 4.4K and 300K for Co and Dy are listed in Fig1.(e), (f), (g) and (h), respectively. Thus magnetic spin, orbital moments of Dy and Co at 4.4K and 300K are obtained and listed in Table.1. For Co the magentic dipole term T_z is neglected. For rare earth of Dy, following [2], $3\langle T_z\rangle$ is considered as $-\frac{2}{3}\langle S_z^{eff}\rangle$.

At 4.4K, Dy dominates the out of plane magnetization since its total magnetic moment is positive and thus parallel pointing into the magnetic field direction while Co magnetic moment is negative and thus antiparallel. At 300K, the spin structure reverses and z-component of Co moments align now in direction of the magnetic field. Magnetic spin and orbital moments for Dy and Co atoms, respectively, are listed for $DyCo_4$ at 4.4K and 300K in Table.1.

TABLE I. Magnetic moments for Dy and 4 Co atoms in $DyCo_4$ at 4.4K and 300K, with $\mu_0H = 6T$.

	$m_s(\mu_B/DyCo_4)$	$m_l(\mu_B/DyCo_4)$	$m_{total}(\mu_B/DyCo_4)$
Co(4.4K)	-4.8±-0.50	-0.50±-0.10	-5.30±-0.50
Dy(4.4K)	4.00±0.30	3.55±0.40	7.55±0.70
Total	-0.80±-0.60	3.05±0.30	2.25±0.85
Co(300K)	5.60±0.60	0.80±0.10	6.40±0.60
Dy(300K)	-2.30±-0.20	-1.80±-0.20	-4.00±-0.30
Total	3.30±0.60	-1.00±0.20	2.40±0.70

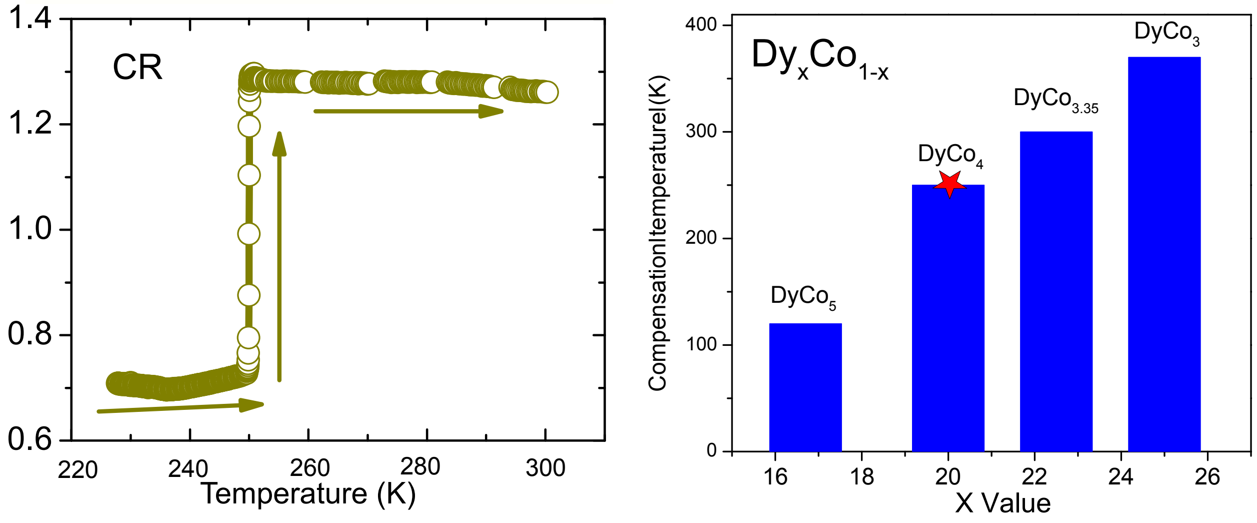

FIG. 2. (a)Temperature dependent bulk CR polarized absorption signal at Dy M_5 with a sharp enhancement at T_{comp} = 250K indicating the spin reveasal, with an applied field of $\mu_0 H = 2T$. (b)T_{comp} for Dy_xCo_{1-x}, $DyCo_5$ from[5], $DyCo_{3.35}$ from [6] and $DyCo_3$ from [7]

2:COMPENSATION TEMPERATURE

The compensation temperature $T_{comp} \sim$ 250K, at which both magnetic contributions from Dy and Co cancel out, is determined for $DyCo_4$ from behavior of the coercivity $\mu_0 H_c$ for the temperature dependent magnetic hysteresis loops measured in the transmission mode (see Fig3.a in the paper). The compensation temperature is determined to be $\sim$ 250K at which the $\mu_0 H_c$ is too high to emerge. Besides, with an applied field of $\mu_0 H = 2T$ and the temperature increasing, CR polarized absorption signal at Dy M_5 increased sharply at 250K which confirmed the spin reveasal of Dy and Co at T_{comp} = 250K. T_{comp} for Dy_xCo_{1-x} are also listed for comparison with the data of $DyCo_5$ from[5], $DyCo_{3.35}$ from [6] and $DyCo_3$ from [7].

3:MAGNETIC HYSTERESIS LOOPS

To investigate the macroscopic behavior of magnetization in more details between both elements, element specific magnetic hysteresis curves were recorded and shown in Fig.3(a) and (b), by measuring the field dependent XMCD signal from transmission at the Co L_3 edge and the Dy M_5 edge at selected temperatures between 4.4K to 300K. The magnetic field sweeps are carried out with the magnetic field aligned in the out-of-plane direction. For all temperatures, the bulk magnetic hysteresis loops of Co and Dy are of the quasi identical shape but opposite sign, indi-

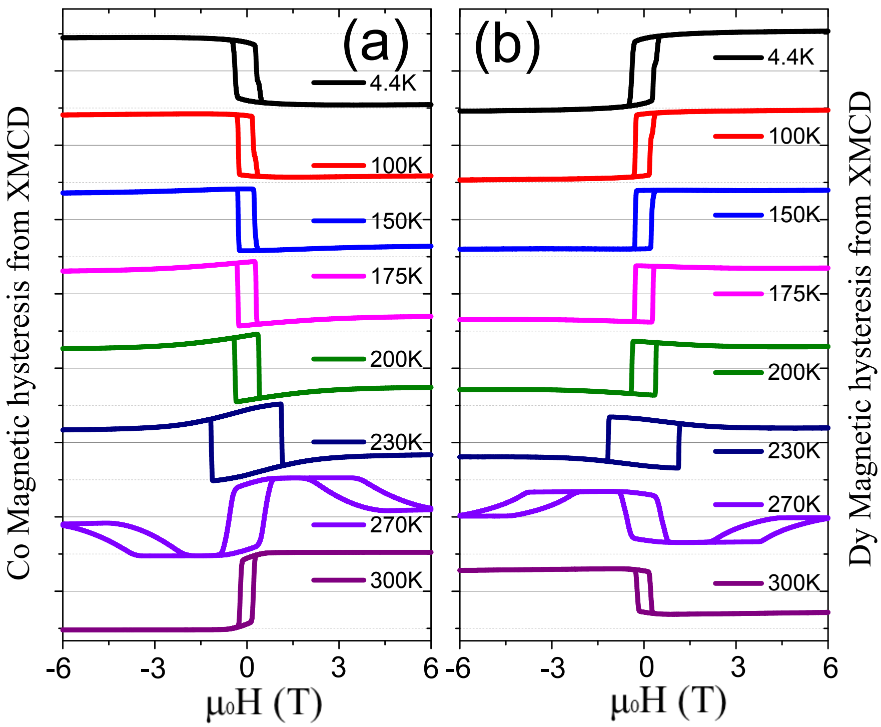

FIG. 3. (color Online) Magnetic hysteresis derived from XMCD for Co (a) and Dy(b) with T from 4.4 to 300K. The bulk magnetic hysteresis lopps of Co and Dy have similar shape but opposite sign, this indicates a ferrimagentic coupling between Co and Dy. The shape of the magnetic hysteresis loops are very sensitive to the temperature for both Co and Dy.

cating a strong ferrimagentic coupling between Co and Dy. The shape of the magnetic hysteresis loops are very sensitive to the temperature for both Co and Dy.

As shown in Fig.4 at T=270K, at both and bulk part, Dy and Co have the opposite magentization according to the magnetic hysteresis. The exchange bias effect is usually defined for net magnetization, thus the magnetic hysteresis loops of the net magnetization $M = M_{Co} + M_{Dy}$ at the surface and bulk part are deduced and provided in Fig.4.

[1] B. T. Thole, P. Carra, F. Sette, and G. van der Laan, Phys. Rev. Lett. 68, 1943 (1992).
[2] P. Carra, B. T. Thole, M. Altarelli, and X. Wang, Phys. Rev. Lett. 70, 694 (1993).
[3] C. T. Chen, Y. U. Idzerda, H.-J. Lin, N. V. Smith, G. Meigs, E. Chaban, G. H. Ho, E. Pellegrin and F. Sette, Phys. Rev. Lett. 75, 152 (1995).

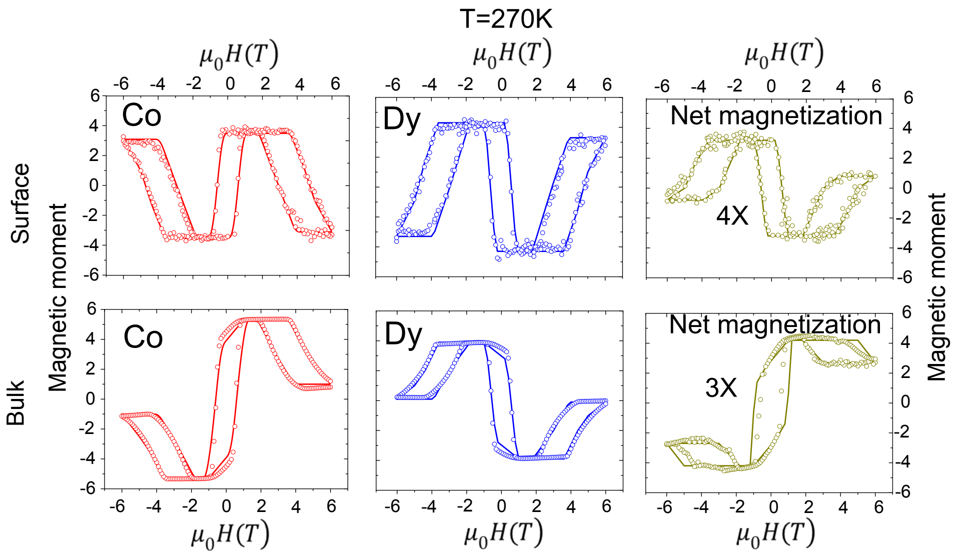

FIG. 4. (color Online) Magnetic hysteresis derived from XMCD for Co and Dy, from surface and bulk part with T=270K, the net magnetization $M = M_{Co} + M_{Dy}$ are also shown (4 or 3 times enlarged for viewing). The surface and bulk hysteresis loops of Co and Dy have similar shape but opposite sign.

[4] J. B. Goedkoop, B. T. Thole, G. van der Laan, G. A. Sawatzky, F. M. F. de Groot, and J. C. Fuggle, Phys. Rev. B 37, 2086 (1988).

[5] F. Radu, R. Abrudan, I. Radu, D. Schmitz and H. Zabel, Nat. Commun. 3, 715 (2012).

[6] A. Agui, M. Mizumaki, T. Asahi, J. Sayama, K. Matsumoto, T. Morikawa, T. Matsushita, T. Osaka and Y. Miura, Journal of Alloys and Compounds. 408-412, 741-745 (2006)

[7] K. Chen,D. Lott, F. Radu, F. Choueikani, E. Otero, and P. Ohresser, Phys. Rev. B, 91, 024409 (2015)
